# The Effect of Thermal History on the Fast Crystallization of Poly(l-Lactide) with Soluble-Type Nucleators and Shear Flow

**DOI:** 10.3390/polym8120431

**Published:** 2016-12-10

**Authors:** Tianfeng Shen, Piming Ma, Qingqing Yu, Weifu Dong, Mingqing Chen

**Affiliations:** The Key Laboratory of Food Colloids and Biotechnology, Ministry of Education, School of Chemical and Material Engineering, Jiangnan University, 1800 Lihu Road, Wuxi 214122, China; stf0710@126.com (T.S.); yqq1835@163.com (Q.Y.); wfdong@jiangnan.edu.cn (W.D.); mq-chen@jiangnan.edu.cn (M.C.)

**Keywords:** poly (l-lactide), crystallization, soluble-type nucleator, shear flow, melting process

## Abstract

The *N*_1_,*N*_1_ʹ-(ethane-1,2-diyl)bis(*N*_2_-phenyloxalamide) (OXA) is a soluble-type nucleator with a dissolving temperature of 230 °C in poly(l-lactic acid) (PLLA) matrix. The effect of thermal history and shear flow on the crystallization behavior of the PLLA/OXA samples was investigated by rheometry, polarized optical microscopy (POM), differential scanning calorimetry (DSC), wide angle X-ray diffraction (WAXD), and scanning electron microscopy (SEM). The crystallization process of the PLLA/OXA-240 sample (i.e., pre-melted at 240 °C) was significantly promoted by applying a shear flow, e.g., the onset crystallization time (*t*_onset_) of the PLLA at 155 °C was reduced from 1600 to 200 s after shearing at 0.4 rad/s for even as short as 1.0 s, while the crystallinity (*X*_c_) was increased to 40%. Moreover, the *t*_onset_ of the PLLA/OXA-240 sample is 60%–80% lower than that of the PLLA/OXA-200 sample (i.e., pre-melted at 200 °C) with a total shear angle of 2 rad, indicating a much higher crystallization rate of the PLLA/OXA-240 sample. A better organization and uniformity of OXA fibrils can be obtained due to a complete pre-dissolution in the PLLA matrix followed by shear and oscillation treatments. The well dispersed OXA fibrils and flow-induced chain orientation are mainly responsible for the fast crystallization of the PLLA/OXA-240 samples. In addition, the shear flow created some disordered α′-form crystals in the PLLA/OXA samples regardless of the thermal history (200 or 240 °C).

## 1. Introduction

Biodegradable and biocompatible poly(l-lactic acid) (PLLA) has recently received more and more attention [1,2]. It is expected to partially solve the environment issues that associated with petrochemical materials [3,4,5]. The main drawbacks of PLLA-based materials are brittleness, low crystallization rate, low heat resistant temperatures due to the glass transition temperature (*T_g_*) of about 55 °C, and low crystallinity after conventional processing. Notably, the low crystallization rate has restricted the application range of PLLA.

One effective approach to speed up the crystallization of PLLA is by applying nucleating agents to reduce the nucleating activation energy and simultaneously promote the heterogeneous nucleation effect to achieve higher crystallinity. Many nucleating agents have been investigated including talc [6], clay [7], carbon nanotubes [8], and organic additives such as poly(vinylidene fluoride), orotic acid, *N*,*N*-ethylene-bis(12-hydroxylstearamide) (EBH), nucleobases, substituted-aryl phosphate salts (TMP-5) and *N*,*N*′,*N*″-tricyclohexyl-1,3,5-benzene-tricarboxylamide (TMC-328), *N*,*N*′-bis(benzoyl) hexanedioic acid dihydrazide (TMC-306), and *N*_1_,*N*_1_ʹ-(ethane-1,2-diyl) bis(*N*_2_-phenyloxalamide) (OXA) [9,10,11,12,13,14,15,16,17,18,19,20,21,22]. Among these nucleating agents, TMC-328, TMC-306, and OXA were proven to have high activity and self-assembly ability [18,19,20,21,22]. However, their solubility and self-assembly behavior are temperature and environment (e.g., static or dynamic conditions) dependent. As a consequence, it is still a challenge to optimize the nucleation effect of the self-organized nucleators.

Another approach to speed up the crystallization of semi-crystalline polymers is by using shear flow which can make polymer chains orientate along the flow direction, resulting in plenty of row nuclei, that thereby enhances crystallization kinetics significantly [23,24]. The flow-induced crystallization was mainly applied in polyolefin systems such as poly(ethylene) (PE) and poly(propylene) (PP), and it exists in polymer processing such as injection molding, extrusion, and film blowing [25,26,27]. Although the environmentally friendly PLLA has been commercialized for more than one decade, the flow-induced crystallization of PLLA has just received limited attention compared with polyolefins [28,29]. Furthermore, the effect of nucleating agents in combination with shear flow on the crystallization of PLLA is even less understood [30].

Rheometry was used in the present work to investigate the crystallization behavior of PLLA in the presence of a soluble-type nucleator (OXA) and shear flow. The effect of the thermal history (i.e., melting process) and shear conditions are emphasized and the mechanism of the enhanced crystallization kinetics is discussed. Therefore, the present work not only provides a fundamental research on a complex PLLA/OXA system but also offers a possible approach for high performance PLLA-based products.

## 2. Experimental Section

### 2.1. Materials

Poly(l-lactide) (PLLA, 4032D) was purchased from Nature Works LLC, Minnetonka, MN, USA, with a *M*_n_ = 2.1 × 10^5^ g·mol^−1^, PDI = 1.7, and a d-lactide content of 2%. The nucleating agent, *N*_1_,*N*_1_ʹ-(ethane-1,2-diyl)bis(*N*_2_-phenyloxalamide) (OXA), with a melting temperature of 338 °C and a purity of 98%, was synthesized in the laboratory with the chemical structure as shown in Figure S1.

### 2.2. Sample Preparation

The PLLA and nucleating agent (OXA) were dried at 60 °C in a vacuum oven for 12 h before use. Both of the PLLA/OXA (100/0.5 *wt*/*wt*) and neat PLLA samples were prepared in a chamber of a rheometer (HAAKE Polylab-OS, Thermo Fisher Scientific, Bremen, Germany), at 180 °C and 50 rpm for 5 min. Each sample was then compression molded at 180 °C and 10 MPa for 2 min using a hot compression molding machine and subsequently cooled down with room-temperature compression plates at a pressure of 5 MPa to make disk-shaped samples with a diameter of 25 mm and a thickness of 1.0 mm. The disk-shaped samples were further dried in vacuum at 60 °C for 12 h before the rheological measurements.

### 2.3. Characterization

**Differential Scanning Calorimetry (DSC):** The crystallization and melting behaviour of the samples were studied by using DSC (DSC 8000, Perkin Elmer, Waltham, MA, USA). Each sample was heated to 200 °C at 10 °C/min, held for 3 min at 200 °C, then cooled to 0 °C and re-heated to 200 °C at 10 °C/min. The crystallinity of PLLA (*X*_c_) is calculated via $X_{c}=\left(\Delta H_{m}-\Delta H_{cc}\right)/\left(\omega *\Delta H_{m}^{0}\right)\times 100\%$ [31], where *ΔH*_m_ and *ΔH*_cc_ are the measured melt and cold crystallization enthalpy of the PLLA, respectively, ω is the weight fraction of the PLLA in the blends, and $\Delta H_{m}^{0}$ = 93.6 J/g is the melting enthalpy of 100% crystalline PLLA [32]. All tests were carried out in a nitrogen atmosphere.

**Polarized optical microscopy (POM):** The crystal morphology of the PLA/OXA samples upon cooling from the melt (200 and 240 °C, respectively) were monitored with a POM (Axio Scope 1, Carl Zeiss, Oberkochen, Germany) in combination with a Linkam THMS600 hot-stage. Each sample was sandwiched between two carefully cleaned glass slides and was first held at 200 or 240 °C for 3 min and then cooled to room temperatures at 10 °C/min. Images were taken at varied temperatures.

**Figure 1:**
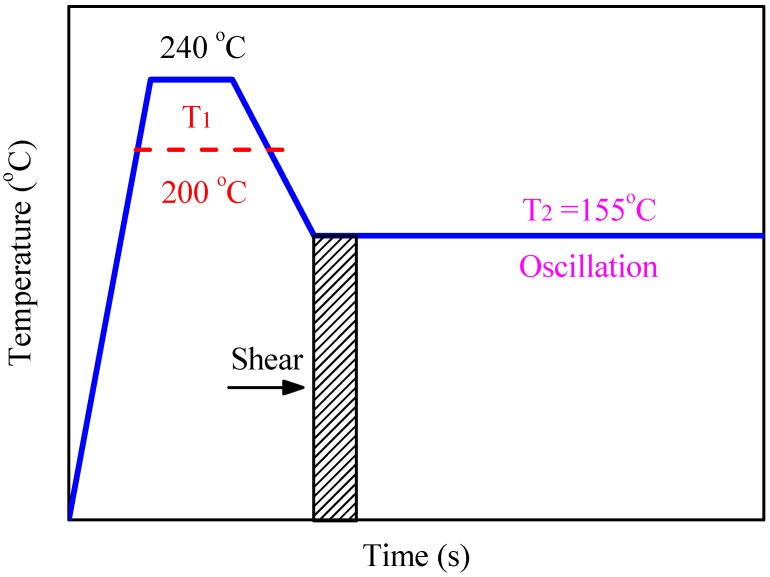
Procedure indications of thermal and shear applications for the rheological measurement of the PLLA/OXA samples.

**Rheology:** Rheological experiments were carried out on a DHR-2 rheometer (TA Instruments, New Castle, DE, USA) in a plate-plate configuration (25 mm in diameter and 1 mm in gap) to study the isothermal crystallization of the PLLA and PLLA/OXA samples with and without pre-shear treatment. The experimental procedures for the shear-induced crystallization are illustrated in Figure 1 and were performed as follows: (1) the samples were annealed at 200 or 240 °C (*T*_1_) for 3 min; (2) subjected to a dynamic temperature sweep with a ramp of −5 °C/min to the desired crystallization temperature (*T*_2_ = 155 °C); (3) a shear pulse with controlled shear rates and shear time was applied on each sample; (4) an oscillatory time sweep was performed at *T*_2_ to trace the evolution of the storage modulus of the samples upon the isothermal crystallization. The strain and frequency were set at 1% and 1 Hz, respectively for the oscillatory time sweep.

The PLLA/OXA samples treated at 200 and 240 °C (*T*_1_) are abbreviated as PLLA/OXA-200 and PLLA/OXA-240, respectively.

**Wide Angle X-ray Diffraction (WAXD):** WAXD measurements were carried out by using an X-ray diffractometer (Bruker AXS D8, Karlsruhe, Germany) equipped with a Ni-filtered Cu Kα radiation source and with a wavelength of 1.542 Å. The measurements were operated at 40 kV and 40 mA with scan angles from 5 ° to 50 ° and a scan rate of 3°/min.

**Scanning Electron Microscopy (SEM):** The micro-morphology of the sheared PLLA/OXA samples was observed by using a SEM (S-4800, Hitachi, Tokyo, Japan) at an accelerating voltage of 2 kV. The cross sections of each sample obtained by cryo-fracture were etched with a 1:2 water-methanol mixture containing 0.025 mol/L NaOH and were subsequently coated with a thin gold layer before observation.

## 3. Results and Discussion

### 3.1. Effect of OXA on the Crystallization of the PLLA under Static Conditions

**Figure 2:**
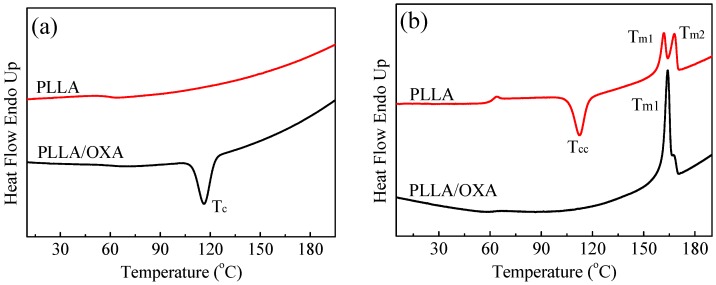
Differential scanning calorimetry (DSC) curves of the PLLA and PLLA/OXA samples: (**a**) cooling from the melt and (**b**) subsequent heating processes. The cooling and heating rates are 10 °C/min.

The effect of OXA on the non-isothermal crystallization and melting behavior of PLLA was studied with DSC, as shown in Figure 2. The corresponding thermal parameters are provided in the Supporting Information (Table S1). No crystallization traces of the PLLA were detected upon cooling (Figure 2a) followed by a pronounced cold crystallization peak in the subsequent heating scan (*T*_cc_ = 112.4 °C, *ΔH*_cc_ = −28.4 J/g, Figure 2b). Huneault et al. reported a *T*_c_ of 103.2 °C for PLLA with talc as a nucleating agent (cooling at 10 °C/min) [6], while Nam et al. reported a *T*_c_ of 110 °C for EBH-nucleated PLLA (cooling at 2 °C/min) [10]. These results demonstrate a poor crystalline capability of PLLA due to chain stiffness and the lack of efficient nucleators [33,34]. A high *T*_c_ and a sharp crystallization peak correspond to a high crystallization rate. Therefore, the DSC data in Figure 2 indicate that the OXA could speed up the crystallization of the PLLA with a narrow crystallization peak (*T*_c_ = 116.2 °C, *X*_c_ = 34.3%). A multi-melting peak behavior can be resulted from different crystalline forms or the same crystalline forms with different perfections. It is reported that PLLA can crystallize in three different forms depending on the crystallization conditions (α, β, γ forms) [35,36,37,38,39]. The α form is the most common in PLLA, while β and γ forms can occur due to special processing conditions. Actually, the β and γ forms should not exist in the present samples as indicated by the XRD results (see below with shear-0). Therefore, a double melting peak of PLLA may be associated with the different crystallization conditions between the α and α′ (also noted as δ) crystals. When PLLA was crystallized at temperatures that were more suitable for α′ crystal formation (e.g., around *T*_cc_ in this work), metastable α′ crystals were formed, and parts of them transformed into stable α crystallites with a higher melting temperature upon heating, leading to the double melting behaviors in the second run of DSC [35].Thus, the double endothermic peaks of PLLA in this work are assigned to a melting/re-crystallization/re-melting mechanism. The *T*_m1_ of PLLA/OXA is slightly higher than that of PLLA (Figure 2b) indicating a better organization and uniformity of PLLA crystals in the presence of OXA.

**Figure 3:**
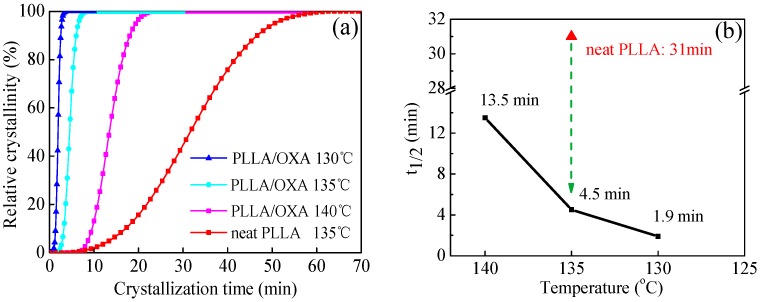
(**a**) The relative crystallinity (*X*_t_) of PLLA and PLLA/OXA as a function of crystallization time (*t*), and (**b**) the corresponding half-life crystallization time (*t*_1/2_) obtained at *X*_t_ = 50%.

The effect of OXA on the isothermal crystallization of PLLA was studied in the temperature range of 130–145 °C. Figure 3 shows the relative crystallinity (*X*_t_) as a function of crystallization time (*t*) and the half-life crystallization time (*t*_1/2_) as a function of temperature (*T*_c_). The crystallization time of the PLLA/OXA sample is shorter in comparison with that of the PLLA. Taking *T*_c_ = 135 °C as an example, the *t*_1/2_ of neat PLLA was 31 min in comparison with 4.5 min of the PLLA/OXA sample. These results indicate that the OXA significantly accelerated the isothermal crystallization process of PLLA. On the other hand, the *t*_1/2_ of PLLA/OXA increased with increasing *T*_c_ because of the difficulty in nucleation at high(er) temperatures. It has to be noted that no crystallization of PLLA/OXA occurred within 90 min at 145 °C (see Figure S2). In literature, crystallization of PLLA was not observed at 146 °C even in the presence of poly (d-lactic acid) as a nucleating agent [40]. Thus, it would be more difficult for the crystallization of PLLA/OXA at a temperature higher than 145 °C.

### 3.2. Self-Organization of the OXA upon Cooling from Different Temperatures

**Figure 4:**
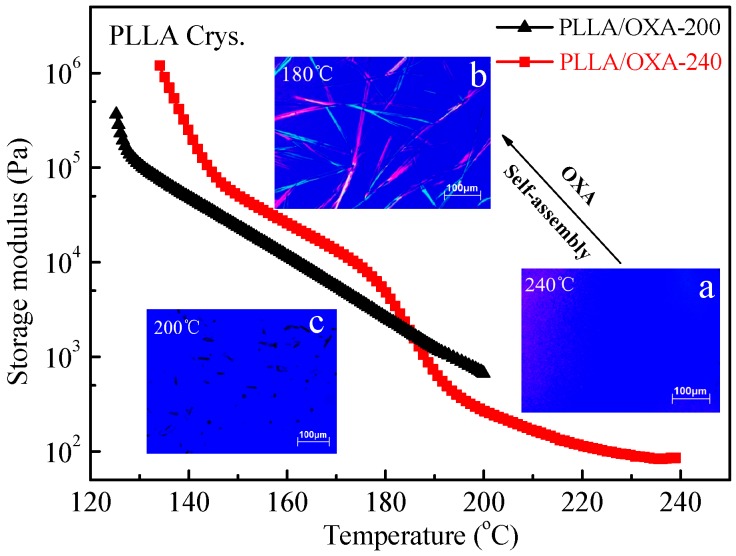
Storage modulus versus temperature for the PLLA/OXA samples upon cooling from 200 and 240 °C, respectively. Three POM images performed under static conditions are shown: (**a**) PLLA/OXA-240 at 240 °C; (**b**) PLLA/OXA-240 at 180 °C; and (**c**) PLLA/OXA-200 at 200 °C.

Since rheological responses are sensitive to microstructural changes [41,42], they are used to investigate the self-organization behavior of OXA in the PLLA melt and the crystallization behavior of PLLA. It is known that the dissolution temperature of OXA in the PLLA melt is around 230 °C [22]. Figure 4 shows the variation of the storage modulus (*G′*) of the PLLA/OXA samples upon cooling from 200 and 240 °C, respectively. For the PLLA/OXA-240 sample, two steep increases in storage modulus are observed around 195 and 145 °C, respectively. Two polarized optical microscopy (POM) images of the PLLA/OXA-240 samples taken upon cooling are presented as insets in Figure 4a,b, which clearly confirmed the self-organized OXA fibrillar superstructures. Therefore, the strong increase at 195 °C is associated with the self-organization process of the dissolved OXA into a non-soluble fibrillar network, while the increase at 145 °C corresponds to the crystallization of the PLLA matrix. Similar phenomena were observed in PLA/TMC-306 systems as well [18]. In the case of the PLLA/OXA-200 sample, an inconspicuous increase of *G′* occurred at around 190 °C followed also by a strong increase around 125 °C. As OXA could only be partially dissolved in the PLLA matrix at 200 °C (Image c), the former increase of *G′* associated with the self-organization of some dissolved OXA is not obvious. The increase at 125 °C also resulted from the crystallization of the PLLA matrix. It is noticed that the crystallization temperature of the PLLA/OXA-240 sample is 20 °C higher than that of the PLLA/OXA-200 sample. Apparently, the melting process is an important factor in the crystallization of PLLA/OXA, which is studied further in the presence of shear flow (see below).

### 3.3. Effect of the Melting Process and Shear Flow on the Crystallization Behaviors of the PLLA/OXA Samples

**Figure 5:**
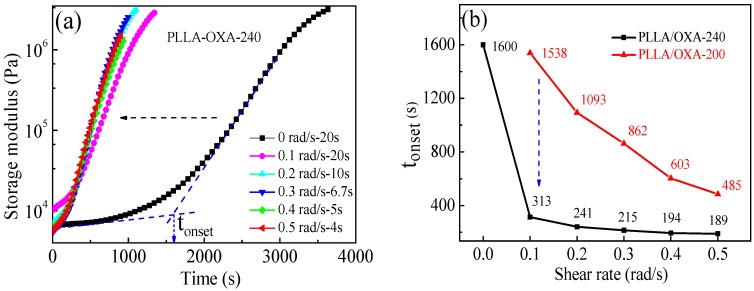
(**a**) Storage modulus of the PLLA/OXA-240 samples as a function of the crystallization time at 155 °C and the shear rate; (**b**) the *t*_onset_ values of the PLLA/OXA samples as a function of the shear rate.

**Effect of shear rate**. Figure 5a shows the *G*ʹ evolutions of the PLLA/OXA-240 samples during the shear-induced isothermal crystallization at 155 °C. A series of shear rates (γ = 0.1, 0.2, 0.3, 0.4, and 0.5 rad/s) were examined while the overall shear angle was fixed at 2 rad by adjusting the shear time. The data for the non-sheared sample (γ = 0.0 rad/s) is plotted for comparison in Figure 5a. For the non-sheared sample, the *G*ʹ slowly rose with time. Impressively, the curve of *G*ʹ~time of the PLLA/OXA-240 samples shifted to a shorter time side rapidly when a shear was applied, regardless of the shear rate. These results indicate a faster overall crystallization rate of the PLLA/OXA sample after melting at 240 °C and shearing at 155 °C.

The inflection point of *G*ʹ~time is defined as the onset crystallization time (*t*_onset_), which is plotted as a function of shear rate for the PLLA/OXA samples that cooled from both temperatures, as shown in Figure 5b. The *t*_onset_ of the PLLA/OXA-240 samples is reduced from 1600 s to around 300 s by increasing the shear rate from 0 to 0.1 rad/s and then leveled off, indicating a remarkable acceleration of the crystallization kinetics. The acceleration is mainly contributed by the promoted nucleation process because the crystallization was performed at the same temperature (155 °C) and the crystal growth rate of a polymeric material is usually the same at a certain temperature. Figure 5b also shows that the *t*_onset_ of the PLLA/OXA-240 samples is much shorter than that of the PLLA/OXA-200 samples at the same shear rate, and the differences between the *t*_onset_ values of the PLLA/OXA-240 and the PLLA/OXA-200 samples are larger at lower shear rates. The non-sheared PLLA/OXA-200 sample did not show inflection point (*t*_onset_) within a couple of hours (data are not shown here). Therefore, it can be concluded from these results that a high(er) melting temperature (e.g., 240 °C) and a shear flow are both beneficial to the fast crystallization of PLLA/OXA samples.

The crystallized PLLA/OXA-240 samples were collected after the rheological experiments for differential scanning calorimetry (DSC) characterization, shown in Figure S3 and Table S2. It was found that the crystallinity of the PLLA increased monotonically with increasing shear rate, while the melting temperature remained constant.

**Figure 6:**
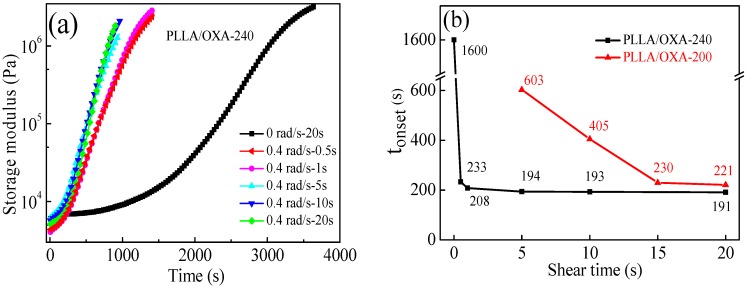
(**a**) Storage modulus of the PLLA/OXA-240 samples as a function of shear time and isothermal crystallization time at 155 °C and (**b**) the *t*_onset_ values of the PLLA/OXA samples as a function of shear time. The shear rate is fixed at 0.4 rad/s for all samples and the PLLA/OXA-200 sample without shear did not crystallize within the experimental time span (90 min).

**Effect of shear time.** A shear rate of 0.4 rad/s was selected to further study the effect of shear time on the crystallization of the PLLA/OXA samples. Figure 6a shows the variations of *G*ʹ of the PLLA/OXA-240 samples as a function of the shearing time and the crystallization time at 155 °C. The corresponding *t*_onset_ as a function of shear time is plotted in Figure 6b. The sheared sample (0.4 rad/s for 0.5 s) shows a much sharper rise of *G*ʹ in comparison with the non-sheared sample, demonstrating a rapid overall crystallization process after the shear. However, the *G*ʹ~time curves did not shift any more when the shear time was longer than 5 s. It implies that the effective orientation of microstructures might become dynamically balanced after a critical shear time (*t*_c_). The *t*_c_ value was 1–5 s for the PLLA/OXA-240 sample at a shear rate of 0.4 rad/s. The *G*ʹ~time of the PLLA/OXA-200 samples showed a similar trend as a function of shear time (data are not shown here), whereas the *t*_c_ value was 15–20 s at the same shear rate.

Similar to the effect of the shear rate, the *t*_onset_ of the PLLA/OXA-240 samples reduced by ~90% when a shear time of 1 s was applied, and then leveled off (Figure 6b). In comparison, the PLLA/OXA-200 sample had a much longer *t*_onset_ value, notably with a short(er) shear time. Taking a shear time of 5 s as an example, the *t_ons_*_et_ of the PLLA/OXA-200 sample was 2 times larger than that of the PLLA/OXA-240 sample. These results further confirm that a higher melting temperature in combination with a shear flow can more effectively promote the crystallization process of PLLA.

### 3.4. Crystal Structure and Morphology of the PLLA/OXA Samples

**Figure 7:**
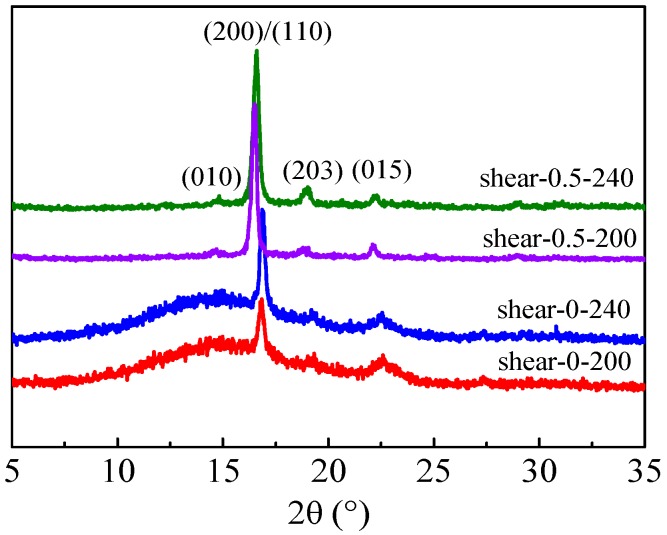
X-ray diffraction patterns of the PLLA/OXA-200 and PLLA/OXA-240 samples with and without a shear flow (0.4 rad/s for 5 s) at 155 °C. The samples were taken after the rheology measurements.

In order to gain deeper insight into the effect of the melt process and shear flow on the crystallization of PLLA, wide angle X-ray diffraction (WAXD) measurements were carried out and the diffraction patterns are shown in Figure 7. For the non-sheared PLLA/OXA samples, three diffraction peaks at 2θ = 16.7°, 19.2°, and 22.6° were detected, correlating to the 200/110, 203, and 105 planes of PLLA α form crystals, respectively [35,43,44]. Meanwhile, a broad diffraction peak was observed for both of the non-sheared PLLA/OXA-200 and PLLA/OXA-240 samples, indicating an incomplete crystallization of the PLLA phase. The peak intensity of the PLLA/OXA-240 sample at 2θ = 16.7° was larger than that of the PLLA/OXA-200 sample, indicating a relatively higher crystallinity of the PLLA/OXA-240 sample. Intriguingly, the diffraction peaks were elevated when a shear flow was applied, accompanied by the disappearance of the broad diffraction peak. Meanwhile, the diffraction peaks of the PLLA/OXA samples shifted by 0.3° to smaller 2θ positions after applying the shear flow regardless of the melting temperatures (*T*_1_ = 200 or 240 °C). A similar result was observed in nucleator-modified PLLA fibers where the shift was ascribed to the existence of some disordered α′-form crystals [45,46]. It has been proven that α- and α′-form crystals share the same 10_3_ helix chain conformation and orthorhombic unit cell, but the packing of the side groups in the helical chains of the α′-form crystals is less ordered and looser than that of the α-form crystals [36].

**Figure 8:**
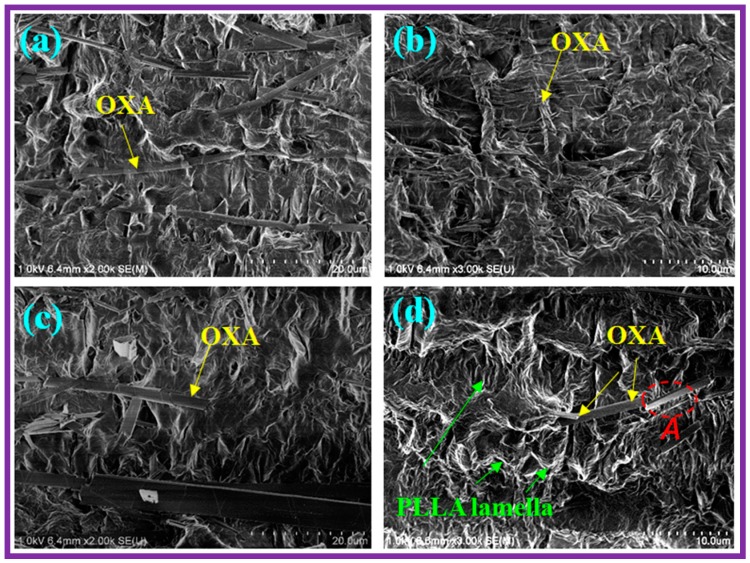
SEM images of the PLLA/OXA samples: (**a**) PLLA/OXA-200 just after shear; (**b**) PLLA/OXA-200 after shear and crystallization; (**c**) PLLA/OXA-240 just after shear and (**d**) PLLA/OXA-240 after shear and crystallization. The same shear condition, i.e., 0.4 rad/s for 5 s was applied to each sample and the crystallization is performed under oscillation conditions. A larger magnification of Images (**b**,**d**) (3000×) was used compared with that of Images (**a**,**c**) (2000×), for better visualization purposes.

The microstructures of the sheared PLLA/OXA samples taken from different crystallization periods were studied using SEM, as shown in Figure 8. The OXA molecules in the PLLA melt can self-organize into fibrils via hydrogen bonding, and are capable of serving as nucleating agents [21]. The self-organized fibrils reassembled into larger needle-like superstructures after a shear flow, as shown in Figure 8a,c. It was observed that the OXA in the PLLA/OXA-240 sample showed better self-organization and alignment in comparison with that in the PLLA/OXA-200 sample. These superstructures were subsequently re-dispersed into even smaller fibrils due to the oscillation effect during the subsequent isothermal crystallization process (Figure 8b,d). The better dispersed OXA fibrils provided extra nucleating sites for the accumulation of PLLA crystals, leading to a type of shish-kebab crystal morphology (area A in Figure 8d). Moreover, the PLLA/OXA-240 sample showed more and thicker PLLA crystals in comparison with the PLLA/OXA-200 sample (Figure 8b,d).

### 3.5. Mechanism Discussion

**Figure 9:**
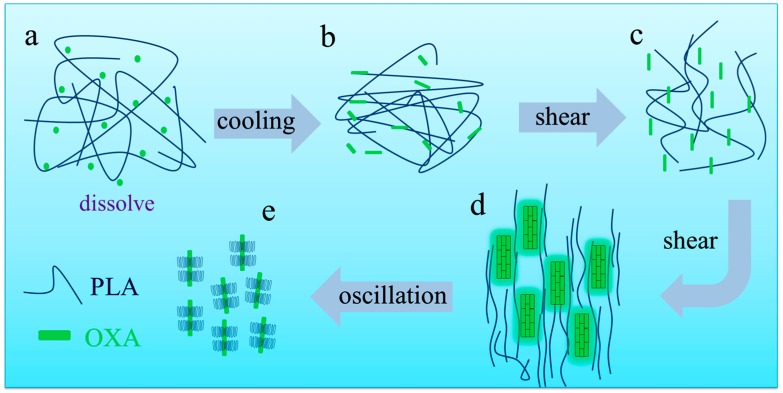
Schematic illustration of the enhanced crystallization kinetics of the PLLA/OXA-240 systems showing the evolution of the OXA superstructures and the orientation of the PLLA macromolecules in the presence of shear flow. (**a**) PLLA/OXA melt with dissolved OXA molecules; (**b**) self-organization of OXA in the PLLA melt; (**c**,**d**) the OXA fibrils and PLLA molecules gradually orientate in the shear direction; (**e**) crystallization of PLLA.

The above results clearly show that the PLLA/OXA-240 samples have better crystallization ability than the PLLA/OXA-200 sample under the same shear and crystallization conditions, and are also better than the non-sheared PLLA/OXA-240 sample. Therefore, a schematic illustration is provided for the mechanism discussion of shear flow induced crystallization of the PLLA/OXA-240 system (Figure 9). At 240 °C, the OXA can be melted and well dissolved in the PLLA matrix, as confirmed by Figure 4a and illustrated in Figure 9a. Upon cooling from 240 to 155 °C, the dissolved OXA molecules can self-organize more homogeneously than the original OXA aggregates (e.g., cooling from 200 °C) into fibrils/needle-like superstructures that are capable of serving as nucleating sites which, however, were randomly distributed. At high temperatures (e.g., 155 °C), PLLA without shear is difficult to crystallize even in the presence of the OXA fibrils [20,21,22]. However, the fibrils and PLLA molecules gradually orientate in the shear direction when a shear flow is applied, see Figure 9c,d. The fibrils superstructures become finer in dimension and are better dispersed in the PLLA matrix due to the subsequent oscillation of the rheometer, providing more nucleating sites (Figure 9e). It is believed that the oriented polymer chains could assemble into a parallel array and form the precursors of primary nuclei for crystallization [23,24]. Therefore, the crystallization kinetics of the PLLA is promoted both by the evolution of the OXA superstructures and by a certain extent of the PLLA chain orientation.

## 4. Conclusions

*N*_1_,*N*_1_ʹ-(ethane-1,2-diyl)bis(*N*_2_-phenyloxalamide) (OXA) was identified as a soluble-type nucleator for poly(l-lactic acid) (PLLA). In the present work, both OXA and the shear flow were applied to accelerate the crystallization of PLLA, at two different melt annealing temperatures (200 and 240 °C). The effect of melting temperature and shear flow on the crystallization of the PLLA/OXA samples at 155 °C was investigated by using rheometry, polarized optical microscopy (POM), differential scanning calorimetry (DSC), wide angle X-ray diffraction (WAXD) and scanning electron microscopy (SEM). As a result, the crystallization of the PLLA/OXA-240 sample was significantly sped up by even a gentle shear flow, e.g., the onset crystallization time (*t*_onset_) of the PLLA could be reduced by ~90% with a shear flow as small as 0.4 rad, while the crystallinity (*X*_c_) reached 40%.

Compared with the PLLA/OXA-200 sample, the *t*_onset_ of the PLLA/OXA-240 sample was reduced by 60%–80% under the same shear conditions (a total shear angle of 2 rad). Therefore, the higher melting temperature (240 °C) does accelerate the crystallization of PLLA in the presence of OXA and shear flow. OXA can be dissolved completely in the PLLA matrix at 240 °C, while only partially dissolved at 200 °C. A better organization and uniformity of the OXA superstructures can be achieved due to the complete pre-dissolution in the PLLA matrix and a subsequent shear and oscillation treatment. The well dispersed OXA fibrils and shear flow induced PLLA chain orientation are responsible for the fast crystallization of the PLLA/OXA-240 samples. In addition, the X-ray diffraction patterns showed that the shear flow created some disordered α′-form crystals in the PLLA/OXA samples regardless of the melting temperatures (200 or 240 °C). The new findings in this work may be applicable to other OXA-nucleated polymeric systems as well, and thus may expand the application range of OXA and PLLA.

## Supplementary Materials

The following are available online at www.mdpi.com/2073-4360/8/12/431/s1. Figure S1: The chemical structure of the OXA, Figure S2: DSC heat flow as a function of isothermal crystallization time and temperatures for the PLLA/OXA (100/0.5 *wt*/*wt*) samples, Figure S3: First heating DSC curves of the sheared PLLA/OXA-240 samples after the rheological experiments at 155 °C, Table S1: Thermal parameters of the PLLA and PLLA/OXA samples obtained from the DSC cooling and 2nd heating scans, Table S2: Thermal parameters of the PLLA/OXA-240 samples derived from Figure S3.
