# Supplementary material for: The Effect of Thermal History on the Fast Crystallization of Poly(l-Lactide) with Soluble-Type Nucleators and Shear Flow
Source: Polymers (Basel). 2016 Dec 10;8(12):431. doi: 10.3390/polym8120431 (PMC6432256; doi:10.3390/polym8120431)
Supplement: Supplementary file 1 [file polymers-08-00431-s001.pdf]

Supplementary Materials: Effect of Thermal History on the Fast Crystallization of Poly(L-Lactide) with Soluble-Type Nucleators and Shear Flow

Tianfeng Shen, Piming Ma, Qingqing Yu, Weifu Dong and Mingqing Chen

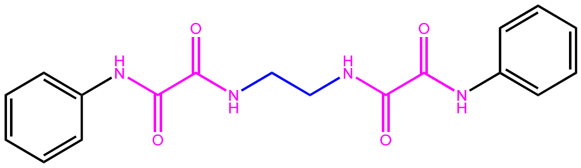

Figure S1. The chemical structure of the OXA.

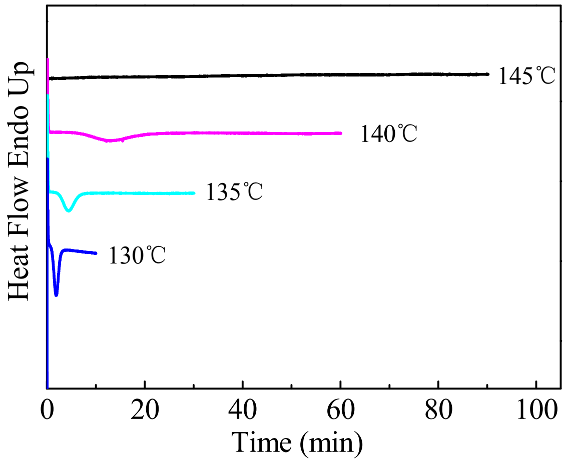

Figure S2. DSC heat flow as a function of isothermal crystallization time and temperatures for the PLLA/OXA (100/0.5 *wt*/*wt*) samples.

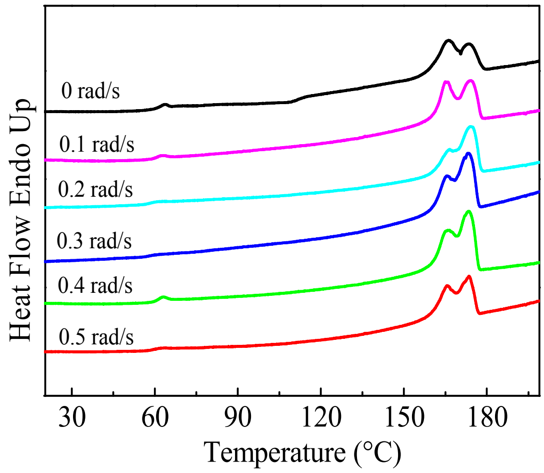

Figure S3. First heating DSC curves of the sheared PLLA/OXA-240 samples after the rheological experiments at 155 °C. The shear rate is different while the overall shear angle is fixed at 2 rad.

Table S1. Thermal parameters of the PLLA and PLLA/OXA samples obtained from the DSC cooling and 2nd heating scans.

Samples	T_c (°C)	ΔH_c (J/g)	T_{cc} (°C)	ΔH_{cc} (J/g)	T_m (°C)	ΔH_m (J/g)	X_c (%)
PLLA	-	-	112.4	28.4	161.9/168.0	28.5	0.0
PLLA/OXA	116.2	32.0	-	-	164.0	32.1	34.3

Table S2. Thermal parameters of the PLLA/OXA-240 samples derived from Figure S3.

PLLA/OXA (rad/s)	T_{m1} (°C)	T_{m2} (°C)	ΔH_m (J/g)	X_c (%)
0.0	166.2	174.0	31.3	33.4
0.1	165.0	174.0	32.8	35.0
0.2	165.1	174.0	37.4	39.9
0.3	165.0	173.4	37.6	40.1
0.4	165.0	173.5	37.9	40.5
0.5	165.1	173.5	38.1	40.7
